# Protective and Curative Effects of the Sea Cucumber *Holothuria atra* Extract against DMBA-Induced Hepatorenal Diseases in Rats

**DOI:** 10.1155/2015/563652

**Published:** 2015-03-02

**Authors:** Ahmed I. Dakrory, Sohair R. Fahmy, Amel M. Soliman, Ayman S. Mohamed, Sayed A. M. Amer

**Affiliations:** ^1^Biology Department, Faculty of Science, Taif University, Saudi Arabia; ^2^Zoology Department, Faculty of Science, Cairo University, Giza 12613, Egypt

## Abstract

Oxidative stress is a common mechanism contributing to the initiation and progression of hepatic damage. Hence there is a great demand for the development of agents with potent antioxidant effect. The aim of the present study is to evaluate the efficacy of *Holothuria atra* extract (HaE) as an antioxidant against 7,12-dimethylbenz[a]anthracene- (DMBA-) induced hepatorenal dysfunction. Experimental animals were divided into two main groups: protective and curative. Each group was then divided into five subgroups pre- or posttreated either with distilled water (DMBA subgroups) or with HaE (200 mg/kg body weight) for seven and fourteen days. Single oral administration of DMBA (15 mg/kg body weight) to Wistar rats resulted in a significant increase in the serum liver enzymes and kidney function's parameters. DMBA increased level of liver malondialdehyde (MDA), decreased levels of reduced glutathione (GSH), glutathione-S-transferase (GST), superoxide dismutase (SOD), and catalase (CAT) in the liver tissue, and induced liver histopathological alterations. Pre- or posttreatment with HaE orally for 14 days significantly reversed the hepatorenal alterations induced following DMBA administration. In conclusion, HaE exhibits good hepatoprotective, curative, and antioxidant potential against DMBA-induced hepatorenal dysfunction in rats that might be due to decreased free radical generation.

## 1. Introduction

Polycyclic aromatic hydrocarbons (PAHs) and their alkylated derivatives are harmful pollutants ubiquitously present in the environment [1]. PAHs are widely distributed as environmental pollutants that are analyzed frequently and have both anthropogenic and natural sources [2]. PAHs are semivolatile organic compounds and present in both gaseous and particulate phases. The partitioning of PAH compounds between the particulate and gaseous phases depends on the atmospheric conditions, the nature of aerosol, the interactions between the compound and the aerosol, and the overall behavior of the compound in the atmosphere [3].

Polycyclic aromatic hydrocarbons, such as 7,12-dimethylbenz[a]anthracene (DMBA), are environmental pollutants that exert multiple toxic and carcinogenic effects [4]. DMBA is an exogenous hepatotoxin, which is well known for modulating phases I and II and antioxidative enzymes of liver [5]. DMBA is a major breast cancer risk [6]. Excessive reactive oxygen species (ROS) are also generated during metabolic activation of DMBA [5]. Emerging evidences suggest that DMBA induces the production of ROS that result in lipid peroxidation, DNA damage, and depletion of cell antioxidant defense systems [5, 7]. Changes in lipid peroxidation production reactions and antioxidant defense systems were associated with changes in a variety of biochemical pathways [8]. DMBA-induced experimental carcinogenesis might therefore be used as an ideal model to study the chemopreventive potential of natural entities [9].

Although there are many strategies for the treatment of liver cancer, the therapeutic outcome of this cancer remains very poor. In spite of tremendous advances in modern medicine, there are not many effective drugs available that stimulate liver function, offer protection to the liver from damage, or help to regenerate hepatic cells [10]. As a consequence of an increasing demand for the therapeutic drugs, products from marine sources have become attractive as nutraceutical and functional foods and as a source material for the development of drugs [11]. Sea cucumber (*Holothuria*) is a gelatinous marine resource that is shaped like a cucumber. It is considered as “sea ginseng” because of its known medicinal properties aside from its nutritional value. The therapeutic use of the sea cucumbers for healing is established, where they were used for joint pain, tendonitis, and sprains [12]. Another report has demonstrated the antinociceptive property of gamat (*Holothuria *spp.) [13]. They are also remarkably rich in vitamins, trace elements, and polysaccharides (chondroitin sulfate), which reduce arthritis pain, inhibit viral activities, and saponin glycosides that inhibit cancer activities [14]. Recently, Esmat et al. [15] revealed that the sea cucumber mixed extract contains physiologically active phenolic compounds with antioxidant activity, which afforded a potential hepatoprotective activity against thioacetamide induced liver injury in a rat model.

*Holothuria atra *is the most important and abundant sea cucumber species in the Red Sea on the Saudi Arabia coast [16]. The current study aims to evaluate the protective and curative effects of the sea cucumber* Holothuria atra* extract (HaE) against the polycyclic aromatic hydrocarbon DMBA-induced hepatorenal diseases in male Wistar albino rats.

## 2. Materials and Methods

### 2.1. Sample Collection

Sea cucumbers (*Holothuria atra*) were collected from Thuwal area, Saudi Arabia's Red Sea coast. The taxonomic identity of the samples was confirmed based on the studies of Purcell et al. [17]. The animals were transported to our laboratory in an ice box containing ice cubes and a few pinches of table salt. The animals were immediately washed under running tap water and cut open, and all visceral organs were removed. The animals were rinsed thoroughly of any internal organs or body fluids, and then the body walls of the animals were stored at –20°C until processing.

### 2.2. Preparation of the* Holothuria atra* Extract (HaE)

The phosphate buffer extract was prepared according to the method of Yasumoto et al. [18]. The body walls of the animals were cut into small parts and blended in phosphate buffer (in a volume = 4 × tissue weight) and extracted at room temperature (25°C) with pH 7.2 for 5 hours. The filtrate was collected immediately, concentrated, and lyophilized using LABCONCO lyophilizer (shell freeze system, USA).

### 2.3. High-Performance Liquid Chromatography Analysis

The phenolic components of the sea cucumber extract were separated by high-performance liquid chromatography using an Agilent 1100 device (Waldborn, Germany) equipped with a Zorbax reversed-phase 300SB C18 column (250–4.6 mm) with 5 mm particle size (Lawrence, KS, USA) and ultraviolet detector (G1314A) adjusted at 280 nm. Sample and authentic standards (50 mL; chlorogenic acid, coumaric acid, catechin, ascorbic acid, pyrogallol, and rutin) dissolved in dimethyl sulfoxide and acidified with a drop of acetic acid were injected onto the column. The mobile phase was 0.4% formic acid and acetonitrile (60 : 40, v/v), with a constant flow rate of 1 mL/min. The isolated peaks of the phenolic compounds in the sample were identified by comparing their relative retention times with those of the standards, and then the concentration (percentage) of each compound was calculated as peak area integration.

### 2.4. Free Radical Scavenging Activity

The free radical scavenging activities of the extract and ascorbic acid were analyzed by the DPPH assay [19]. 1.0 mL of the test extract, at gradient final concentrations of 10–80 mg/mL, was mixed with 2 mL of 0.3 mM DPPH solution in MeOH in a cuvette. The absorbance was taken at 517 nm after 20 minutes of incubation in the dark at room temperature. The experiment was done in triplicate. The percentage antioxidant activity was calculated as follows:

(1) %Antioxidant  Activity AA  =100−Abssample−Absblank×100Abscontrol,

where Abs_sample_ was the absorbance of sample solution (1.0 mL) + DPPH solution (2.0 mL, 0.3 mM), Abs_blank_ was the absorbance of methanol (2.0 mL) + sample solution (1.0 mL), and Abs_control_ was the absorbance of DPPH solution (2.0 mL, 0.3 mM) + methanol (1.0 mL).

### 2.5. Ethical Consideration

Experimental protocols and procedures used in this study were approved by the Cairo University, Faculty of Science, Institutional Animal Care and Use Committee (IACUC) (Egypt) (CUFS/F/16/14). All the experimental procedures were carried out in accordance with international guidelines for the care and use of laboratory animals.

### 2.6. Experimental Animals

The experimental animals used in this study were male Wistar rats (*Rattus norvegicus*) weighing 150–160 ± 5 g. The animals were obtained from the National Research Center (NRC, Dokki, Giza). Animals were grouped and housed in polyacrylic cages (six animals per cage) in the well-ventilated animal house of the Department of Zoology, Faculty of Science, Cairo University. Animals were given food and water* ad libitum*. Rats were maintained in a friendly environment with a 12 h/12 h light-dark cycle at room temperature (22°C–25°C). Rats were acclimatized to laboratory conditions for 7 days before commencement of the experiment.

### 2.7. Toxicity Study (OECD 420)

Eighteen Wistar rats weighing 150–160 g were used for acute toxicity studies. The animals were divided into control and test groups containing six animals each. The rats were administered orally with sea cucumber* Holothuria atra* extract (HaE) at dose levels of 5 g/kg (high dose) and 2 g/kg (low dose). Normal control rats received the same amount of vehicle (distilled water) only. Animals were observed carefully for 24 hours after extract administration and then for the next 14 days. At the end of this experimental period, the rats were observed for signs of toxicity, morphological behavior, and mortality. Acute toxicity was evaluated based on the number of deaths (if any). Acute toxicity was calculated as per OECD guidelines 420 (fixed dose method) [20, 21]. The effective dose of the HaE will be calculated as 10% of the safety tested dose of OECD test.

### 2.8. Experimental Design

**Figure 1:**
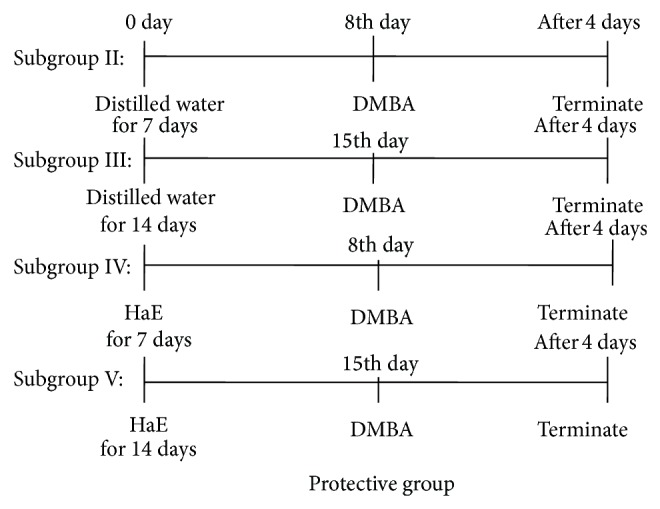
Schematic diagram shows the experimental design of treatment in the protective group.

**Figure 2:**
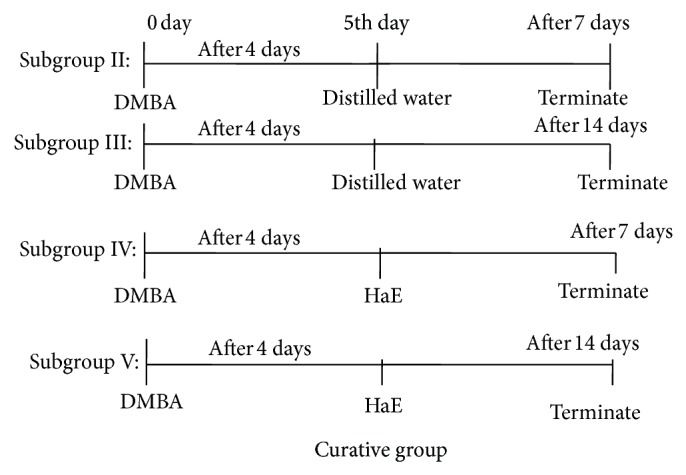
Schematic diagram shows the experimental design of treatment in the curative group.

Sixty male Wistar rats were assigned into two main groups (30 rats/group): the protective (Figure 1) and the curative groups (Figure 2).

*Protective Group*. The animals of this group were divided into five subgroups (6 rats/group) as follows.

 
*Subgroup I*: rats administered distilled water and then challenged with a single oral dose of 1 mL corn oil (control).
 
*Subgroup II*: rats treated with distilled water for 7 days prior to a single dosage of DMBA (15 mg/kg body weight: p.o.) dissolved in 1 mL corn oil on the 8th day of treatment.
 
*Subgroup III*: rats treated with distilled water for 14 days prior to a single dosage of DMBA (15 mg/kg body weight: p.o.) dissolved in 1 mL corn oil on the 15th day of treatment.
 
*Subgroup IV*: rats treated with effective dosage of HaE (200 mg/kg body weight: p.o.) for 7 days prior to a single dose of DMBA (15 mg/kg body weight: p.o.) dissolved in 1 mL corn oil on the 8th day of treatment.
 
*Subgroup V*: rats treated with an effective dose of HaE for 14 days prior to a single dosage of DMBA (15 mg/kg body weight: p.o.) dissolved in 1 mL corn oil on the 15th day of treatment.

The animals were then euthanized 4 days after DMBA administration.

*Curative Group*. The animals of this group were divided into five subgroups (6 rats/group) as follows.

 
*Subgroup I*: rats challenged with a single oral dose of 1 mL corn oil (control) and then administered distilled water.

Animals in subgroups II, III, IV, and V were administered a single dosage of DMBA (15 mg/kg body weight: p.o.) dissolved in 1 mL corn oil and after 4 days were treated as follows.

 
*Subgroup II*: rats treated with distilled water for 7 days.
 
*Subgroup III*: rats treated with distilled water for 14 days.
 
*Subgroup IV*: rats treated with an effective dosage of HaE (200 mg/kg body weight: p.o.) for 7 days.
 
*Subgroup V*: rats treated with an effective dosage of HaE for 14 days.

### 2.9. Animal Handling

Animals were euthanized under sodium pentobarbital. Blood was collected by cardiac puncture in centrifuge tubes. Liver was removed and immediately blotted using filter paper to remove traces of blood and then divided into two parts: the first part was stored at –80°C for biochemical studies, while the second part was suspended in 10% formal saline for fixation preparatory to histological processing.

### 2.10. Sample Preparation

#### 2.10.1. Serum Preparation

Blood samples collected in centrifuge tubes were centrifuged at 860 ×g for 20 minutes. Serum was stored at −20°C until used for biochemical assays.

#### 2.10.2. Liver Homogenate Preparation

Liver tissue was homogenized (10% w/v) in ice-cold 0.1 M Tris-HCl buffer (pH 7.4). The homogenate was centrifuged at 860 ×g for 15 min. at 4°C and the resultant supernatant was used for the biochemical analyses.

### 2.11. Histopathological Preparation

Liver slices were fixed in 10% formal saline and embedded in paraffin wax blocks. Sections of 5 *μ*m thickness were stained with hematoxylin and eosin (H&E) and then examined under light microscope for determination of pathological changes.

### 2.12. Biochemical Assessment

#### 2.12.1. Serum Biomarkers for Liver and Kidney Functions Tests

The appropriate kits (Bio-Diagnostic, Dokki, Giza, Egypt) were used for the determination of serum aminotransferase enzyme activities (AST and ALT) [22]; GGT [23]; total protein [24]; alkaline phosphatase (ALP) [25]; total bilirubin [26]; creatinine [27]; urea; and uric acid [28].

#### 2.12.2. Oxidative Stress Markers Assessment

Oxidative stress markers were detected in the resultant supernatant of liver homogenate. The appropriate kits (Bio-Diagnostic kits, Bio-Diagnostic, Dokki, Giza, Egypt) were used for the determination of malondialdehyde (MDA) [29], reduced glutathione (GSH) [30], catalase (CAT) [31], glutathione-S-transferase (GST) [32], and superoxide dismutase (SOD) [33].

### 2.13. Statistical Analysis

Values were expressed as mean ± SE. To evaluate differences between the groups studied, one-way analysis of variance (ANOVA) with the Duncan post hoc test was used to compare the group means and *P* < 0.05 was considered statistically significant. SPSS for Windows (version 15.0) was used for the statistical analysis.

## 3. Results

### 3.1. Phenolic Compounds in the* Holothuria atra* Extract

**Figure 3:**
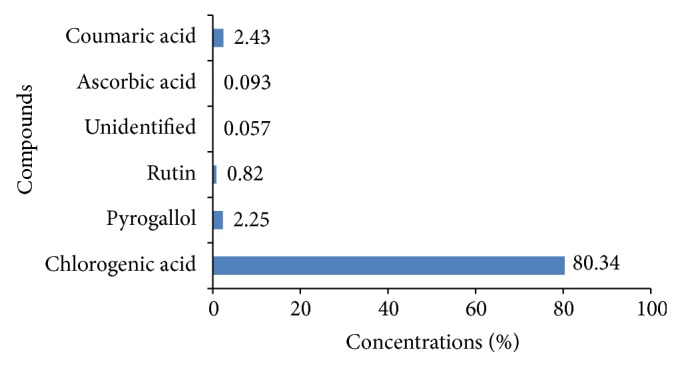
High-performance liquid chromatography analysis of* Holothuria atra* extract (HaE).

Figure 3 revealed that the high-performance liquid chromatography analysis of HaE showed the presence of six nonvolatile phenolic compounds, one of which was unidentified under the adopted conditions. Chlorogenic acid was the major component (80.34%), whereas ascorbic acid (0.093%) was the minor component. Other components, such as pyrogallol (2.25%), rutin (0.82%), and coumaric acid (2.43%), were recorded in Figure 3.

### 3.2. Free Radical Scavenging Activity

**Figure 4:**
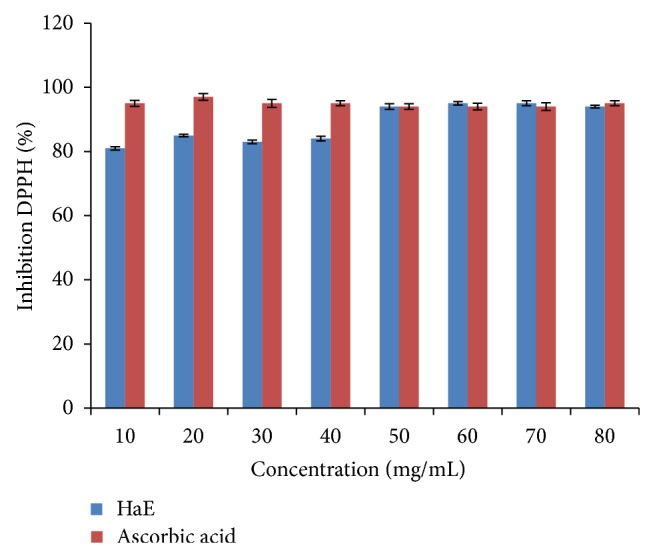
Inhibition of DPPH of* Holothuria atra* extract (HaE).

The radical scavenging activities were estimated by comparing the percentage of inhibition of DPPH radical by the tested extract (HaE) and the ascorbic acid. The data were displayed with mean ± SEM of three replications. The present findings revealed that HaE produced dose dependent inhibition of DPPH radical ranging from 81 to 94% as compared to ascorbic acid (Figure 4).

### 3.3. Toxicity Study (OECD 420)

The present results revealed that the* Holothuria atra *extract (HaE) has been found to be toxic at 5000 mg/kg body weight of experimental animals as assessed by the occurrence of morbidity in the first 6 hours. None of the 6 rats died or showed any sign of toxicity at the limit dose of 2000 mg/kg p.o. for HaE in the first 48 h. No evidence of toxicity was noted during the period of observation. The LD_50_ was therefore taken as above 2000 mg/kg p.o. The median effective dose (ED_50_) was selected based on the proposed LD_50_ obtained from the acute toxicity study. This dose was considered one-tenth of the proposed LD_50_, that is, 200 mg/kg body weight.

### 3.4. Serum Biomarkers for Liver and Kidney Functions Tests

The results of the present study clearly indicate that the* Holothuria atra extract* possesses both protective and curative activities. Tables 1, 2, and 3 illustrate the effect of HaE on some biochemical parameters in the control and treated groups against DMBA-induced hepatic toxicity in male rats. Significant increase (*P* < 0.05) was noticed in the levels of ASAT, ALAT, GGT, creatinine, uric acid, and urea of DMBA intoxicated rats as compared to the corresponding control groups. Conversely, serum total protein level of DMBA treated rats was found significantly decreased (*P* < 0.05), as compared to the corresponding control (Table 2).

Pretreatment of rats with HaE before challenging with DMBA for seven consecutive days caused a significant decrease (*P* < 0.05) only for the serum levels of GGT and urea, as compared to the corresponding DMBA group (Tables 2 and 3). However, a significant decrease (*P* < 0.05) was noticed for all the studied serum parameters subsequent to pretreatment with HaE for 14 days, as compared to the corresponding DMBA group (Tables 1, 2, and 3).

Significant decrease (*P* < 0.05) was observed in the levels of ALAT, GGT, and uric acid of rats posttreated with HaE for seven days, as compared to the corresponding DMBA group, while posttreatment with HaE for 14 days was found to cause a significant decrease (*P* < 0.05) in all the studied serum parameters, as compared to the corresponding DMBA group (Tables 1, 2, and 3).

### 3.5. Oxidative Stress Markers Assessment

There was a general increase in the level of liver MDA subsequent to DMBA intoxication either in protective or in curative groups, as compared to the corresponding control groups (Table 4). On the other hand, liver GSH, GST, SOD, and CAT levels decreased significantly (*P* < 0.05) subsequent to DMBA intoxication either in protective or in curative groups, as compared to the corresponding control groups (Tables 4 and 5).

*Holothuria atra* extract administration (200 mg/kg body weight, p.o.) before or after DMBA administration for 7 days did not cause any significant changes towards the alterations in all the studied oxidative stress markers caused by DMBA intoxication. However, pre- or posttreatment with HaE for 14 days caused significant (*P* < 0.05) decrease in the liver MDA level and an increase in the liver GSH level, GST, SOD, and CAT activities, as compared to the corresponding DMBA intoxicated groups (Tables 4 and 5).

These aforementioned results reflected the efficacy of HaE when used for long time periods either as pretreatments or as posttreatments.

### 3.6. Liver Histopathological Examination

**Figure 5:**
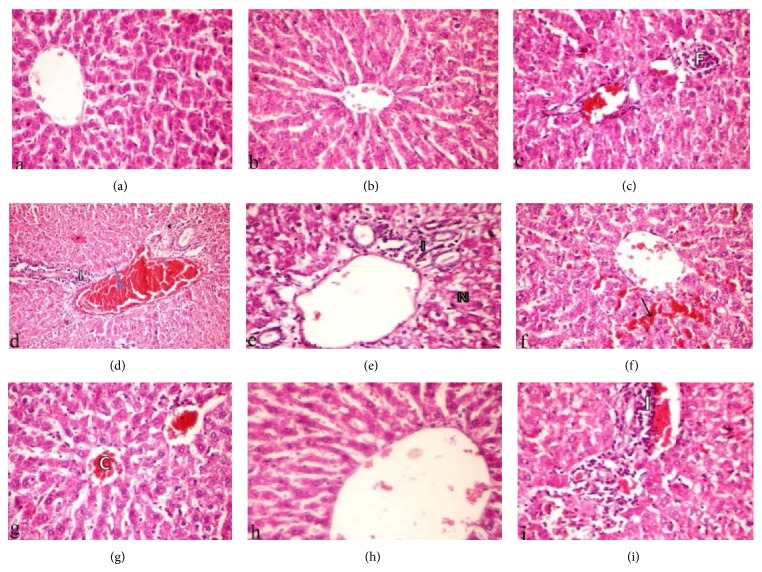
Hematoxylin and eosin stained liver sections from (a) control rats; ((b) and (c)) DMBA intoxicated rats in pre- and posttreatment groups for 7 days; ((d) and (e)) DMBA intoxicated rats in pre- and posttreatment groups for 14 days; (f) 7-day pretreatment rats with HaE; (g) 7-day posttreatment rats with HaE; (h) 14-day pretreatment rats with HaE; (i) 14-day posttreatment rats with HaE (H&E × 400).

Figure 5(a) shows normal histology of the liver, which exhibited the well organized lobular architecture with hepatocyte sand apparently healthy liver parenchyma. DMBA intoxication in pre- and posttreatment groups either before or after 7 and 14 days of distilled water administration caused focal necrosed areas of hepatocytes infiltrated with mononuclear cells (F) (Figures 5(b) and 5(c)), congestion of the hepatoportal blood vessel (C) together with leucocyte cells infiltration (I) (Figure 5(d)), and necrosed hepatocytes (N) and portal leucocytic infiltration (I) (Figure 5(e)).

Pre- and posttreatment with HaE for 7 days caused dilatation and congestion to the blood sinusoids of the liver (arrow) (Figure 5(f)) and congested central vein (C) (Figure 5(g)). However, 14 days of HaE pretreatment could save the liver tissue from DMBA intoxication as the liver showing apparently healthy parenchyma (Figure 5(h)). On the other hand, posttreatment for 14 days could not ameliorate the alterations in liver tissue caused by DMBA intoxication to some extent as liver showing portal tract mononuclear cell infiltrations (I) (Figure 5(i)).

## 4. Discussion

7,12-Dimethylbenz[a]anthracene (DMBA), a member of polycyclic aromatic hydrocarbons (PAHs) class of carcinogens, is present in the environment as a product of incomplete combustion of complex hydrocarbons [34]. The greater susceptibility of liver to damage by chemical agents is presumably a consequence of its primary role in the metabolism of xenobiotics [35]. Exposure to PAHs, including DMBA, can lead to toxicological changes in the liver, including oxidative stress and production of carcinogenic metabolites [36]. DMBA is metabolized by cytochrome P_450_ enzymes in the liver to form diol epoxides and other toxic reactive oxygen species [34]. Oxidation, reduction, and hydrolysis are three important reactions through which phase I metabolism operates [37]. Phase I biotransformation reactions increase the polarity of xenobiotics either by adding or by exposing functional groups and thereby facilitating their excretion from the body.

The modern medicinal system relies heavily on synthetic chemicals being used as drugs, but these unnatural synthetic drugs often pose serious side effects [38, 39]. Therefore, the development of novel chemotherapeutic agents would play a key role in the treatment of many refractory diseases. Many compounds that are derived from marine organisms have generated interest both as challenging problems for structure elucidation and synthesis and for their cytotoxicity [40, 41]. It is believed that a rich source of therapeutic drug candidates could be obtained from marine organisms or their metabolites. The sea cucumber (*Holothuria*) is a marine invertebrate of the phylum Echinoderm and the class Holothuroidea found on the sea floor worldwide [42]. The present study explores the protective and curative roles of* Holothuria atra* extract (HaE) against DMBA-induced changes in hepatic xenobiotic and oxidative enzymes in rats.

It was reported that the presence of the active phenolic compounds in the body wall of the sea cucumbers may be due to phenolic-rich materials such as phytoplankton and particles derived from degrading marine macroalgae which are the main sources of food for sea cucumbers [42]. High-performance liquid chromatography analysis of the phenolic compounds in the HaE revealed the presence of 80.34% of chlorogenic acid. The potential hepatoprotective effect of chlorogenic acid in several animal models of liver injury was reported [43].

In the assessment of liver toxicity by DMBA, the determination of enzyme levels, such as serum ASAT, ALAT, and GGT, is largely used [44–46]. The present study illustrated that DMBA administration elevated AST, ALT, and GGT enzyme activities in the serum of rats. The elevated activities of serum ASAT, ALAT, and GGT observed in DMBA treated group are considered indicative of DMBA-induced hepatic damage [8, 46] and subsequent leakage of these enzymes into circulation. In the present study, DMBA administration resulted in hepatocellular lesions as indicated by focal necrosed areas of hepatocytes infiltrated with mononuclear cells, congestion of the hepatoportal blood vessel together with leucocyte cells infiltration, and necrosed hepatocytes as well as portal leucocytic infiltration.

These findings are in accordance with the results of many other investigators [47, 48]. They observed that DMBA-induced liver-carcinoma in rats indicated by the development of nodules and the liver cells displayed eosinophilic, dense, and pleomorphic nuclei, cytoplasmic vacuolization, and necrosis. Treatment with HaE prior to or after DMBA intoxication for 7 days caused a marked decrease in the levels of serum AST, ALT, and GGT activities; however this decrease was significant following treatment with HaE prior to or after DMBA administration at the tested dose for 14 days indicating maintenance of functional integrity of hepatic cell membrane. Such amelioration of serum enzyme activities could be attributed to the antioxidant properties of the HaE and their ability to scavenge the free radicals hence protecting the cellular membranes integrity from oxidative damage DMBA toxicity.

The major cause of metabolic dysfunction during pathogenesis is oxidative damage of some of the susceptible amino acids of proteins [49]. In accord with the studies of Sharma et al. [50] and El Kholy et al. [45], the present study showed that DMBA intoxication decreased the serum total protein content. It was reported that decline in total protein can be deemed as a useful index of the severity of cellular dysfunction in chronic liver diseases as manifested by the severe histopathological alterations of the liver tissue following DMBA treatment.

This study served to determine potential correlations between DMBA exposure and oxidative stress in the liver, since metabolic activation and detoxification of DMBA* in vivo* occur primarily in this organ [51, 52]. In conjunction with the reports of Parmar et al. [53] and Parmar et al. [54], data from the present investigation reflects that oxidative stress in the liver is a common feature of DMBA toxicity. The present study revealed that DMBA increased malondialdehyde concentration (MDA) in the liver tissue. These results are similar to the data reported by El Kholy et al. [45] and Ahmed et al. [46] who indicated that DMBA intake produced oxidative stress in liver of rats. The increased MDA level suggests enhanced lipid peroxidation leading to tissue damage and failure of antioxidant defense mechanisms to prevent formation of excessive free radicals. Treatment with HaE at tested dose and after 14 days either prior to or after DMBA intoxication significantly reversed these changes, suggesting that the mechanism of HaE hepatoprotection may be due to its antioxidant effect.

Due to its reducing properties, glutathione (GSH), a biologically important tripeptide, is essential for maintaining cell integrity. It is well known that GSH is involved in the protection of normal cell structure and function in maintaining of the redox homeostasis and quenching of free radicals and by participating in detoxification reactions [55]. The present study confirmed the finding of Parmar et al. [53] and El Kholy et al. [45] who suggested that the enhancement of lipid peroxidation is a consequence of depletion of reduced glutathione (GSH) to certain critical levels. The depletion of GSH promotes generation of reactive oxygen species and oxidative stress with a cascade of effects, thereby affecting functional as well as the structural integrity of cell and organelle membranes [56, 57]. Moreover, insufficiency in nonenzymatic antioxidant GSH following DMBA intoxication could be the consequence of increased utilization for trapping free radicals. Treatment with HaE prior to or after DMBA administration for 14 days in the present study increased significantly GSH content in the liver tissue. These findings agreed with the antecedent studies of Gaté et al. [58] and Fahmy and Hamdi [59] who reported that dietary supplementation of the marine extract of the* Crassostrea gigas *clams and* Erugosquilla massavensis *extracts increased GSH level in the liver of rats. The restoration of the GSH level by the HaE could be due to either its effect on the* de novo *synthesis of glutathione, its regeneration, or both [60]. In addition, HaE may act directly and scavenges the ROS derived by oxidation-reduction cycle with the cell or it may work in union with the existing antioxidant compounds and helps to prevent their loss during the oxidative injury caused by DMBA.

Glutathione-S-transferase (GST) isan important phase II enzyme which conjugates reactive metabolites to GSH, resulting in the decrease of its biological reactivity and increases of its solubility for excretion in bile. The present study showed significant decrease in GST in the DMBA treated rats as compared to the control group. In accord with our results, Koul et al. [61] and Lakshmi and Subramanian [62] have reported that the enhanced free radical concentration resulting from the oxidative stress conditions can cause loss of enzymatic activity. Administration of HaE at 200 mg/kg body weight for 14 days in the present study causes significant enhancement in the GST activity. The efficient recovery in GST activity highlights the therapeutic efficacy of HaE in alleviating the DMBA-induced oxidative stress in the liver.

Superoxide dismutase (SOD) and catalase (CAT) act as mutually supportive antioxidative enzymes, which provide protective defense against reactive oxygen species [63]. Viewed in conjunction with the report of Parmar et al. [54], El Kholy et al. [45], and Kumar et al. [5], the inhibition of CAT and SOD activities following DMBA intoxication in the present study may be due to the enhancement of the peroxidation end product MDA, which is known to inhibit protein synthesis and the activities of certain enzymes. Pre- or postadministration of HaE at the tested dose enhanced the activities of CAT and SOD in DMBA-induced liver damage. The enhancement in CAT and SOD activities may be to prevent the accumulation of excessive free radicals and protect liver from DMBA intoxication. In agreement with the report of El Kholy et al. [45] and Kumar et al. [5], the liver cells have innate ability to arouse and maintain defense against oxidant by secreting more antioxidants. HaE may overpower DMBA onslaught by suppressing the formation of ROS and protecting the antioxidant machinery. Moreover, the induction of enzymes by the HaE represents a promising chemopreventive strategy as a bifunctional inducer, along with the enhancement of antioxidant system enzymes which affords protection against cellular damage and inhibits cancer promotion.

Since the kidney is involved in the excretion of many toxic metabolic waste products, particularly the nitrogenous compounds, it would therefore be worthwhile to examine the effects of DMBA on the kidney of treated rats. Plasma levels of urea, creatinine, and uric acid are the biomarkers for nephrotoxicity. Viewed in conjunction of the reports of Singh et al. [57], Suhail et al. [64], and Bedi and Priyanka [65], data from the present investigation reflect that DMBA induced marked alteration on renal functions as manifested by a significant increase in the kidney function markers, serum creatinine, urea, and uric acid. The elevation of the serum urea and creatinine concentrations by DMBA appears to suggest the possible upregulation of protein catabolism and concomitant rise in the synthesis of creatinine that needs to be excreted with urine (formed via the reactions of the urea cycle). Meanwhile, the possibly enhanced production of the reactive oxygen species could be renotoxic consequently impairing the functional capacity of the kidney. Pre- or postadministration of HaE at 200 mg/kg body weight for 14 days in the present study causes significant reduction in the plasma levels of urea, creatinine, and uric acid. The efficient recovery in kidney function parameters highlights the therapeutic efficacy of HaE in alleviating the DMBA-induced nephrotoxicity in the liver.

## 5. Conclusion 

From the results of the present study, it can be concluded that HaE is a useful natural product that can alleviate the hepatorenal toxicity resulting from DMBA hydrocarbon exposure. These could constitute areas of future research. Again, the protective or the curative effects offered by the extract may involve its antioxidant and/or oxidative free radical scavenging activities which are based on the presence of the phenolic-rich materials. Any natural compound with antioxidant properties may help in maintaining health when continuously taken as components of dietary foods, spices, or drugs. The increase in the levels of antioxidant profiles, that is, SOD and catalase, by HaE may be attributed to biological significance in eliminating reactive free radicals that may affect the normal functioning of cells. Again, according to the aforementioned results, the efficacy of HaE appeared when administered for long time periods and preferable as protective agents against polycyclic aromatic hydrocarbons, especially 7,12-dimethylbenz[a]anthracene (DMBA).

## Figures and Tables

**Table 1:** Modulatory influence of *Holothuria atra* extract (HaE) on alanine (ALAT) and aspartate (ASAT) aminotransaminase activities of 7,12-dimethylbenz[a]anthracene (DMBA) treated rats.

Treatment	Groups		ALAT (U/mL)	ASAT (U/mL)
Pretreatment	Control		134.25 ± 5.55^ab^	175.05 ± 6.26^a^
	7 days	DMBA	159.50 ± 9.00^cd^	217.35 ± 6.28^de^
		HaE	151.85 ± 4.40^bcd^	206.75 ± 5.36^bcd^
	14 days	DMBA	177.75 ± 5.93^e^	229.60 ± 3.38^d^
		HaE	141.45 ± 5.12^ab^	189.75 ± 3.48^ab^

Posttreatment	Control		133.85 ± 7.10^a^	175.85 ± 6.25^a^
	7 days	DMBA	165.65 ± 4.49^de^	209.60 ± 7.28^cd^
		HaE	145.70 ± 2.75^abc^	195.4 ± 10.73^bc^
	14 days	DMBA	178.75 ± 5.33^e^	229.75 ± 0.93^d^
		HaE	138.70 ± 3.47^ab^	194.60 ± 5.39^bc^

Values are given as mean ± SEM for 6 rats in each group.

Each value not sharing a common letter superscript is significantly different (*P* < 0.05).

**Table 2:** Modulatory influence of *Holothuria atra* extract (HaE) on gamma glutamyl transferase (GGT) activity and total protein content of 7,12-dimethylbenz[a]anthracene (DMBA) treated rats.

Treatment	Groups		GGT (U/L)	Total protein (mg/dL)
Pretreatment	Control		2.54 ± 0.67^a^	17.54 ± 0.45^d^
	7 days	DMBA	5.21 ± 0.81^de^	12.59 ± 0.74^bc^
		HaE	2.08 ± 0.43^abc^	12.12 ± 0.19^b^
	14 days	DMBA	60.16 ± 17.90^e^	14.616 ± 0.59^c^
		HaE	16.90 ± 6.52^ab^	9.96 ± 0.36^a^

Posttreatment	Control		2.48 ± 0.63^a^	16.86 ± 0.57^d^
	7 days	DMBA	7.64 ± 2.68^de^	12.51 ± 1.01^bc^
		HaE	3.24 ± 0.29^abc^	12.79 ± 0.28^bc^
	14 days	DMBA	67.39 ± 21.26^e^	12.85 ± 0.18^bc^
		HaE	44.60 ± 15.53^ab^	9.42 ± 1.40^a^

Values are given as mean ± SEM for 6 rats in each group.

Each value not sharing a common letter superscript is significantly different (*P* < 0.05).

**Table 3:** Modulatory influence of *Holothuria atra* extract (HaE) on some kidney function parameters of 7,12-dimethylbenz[a]anthracene (DMBA) treated rats.

Treatment	Groups		Creatinine (mg/dL)	Uric acid (mg/dL)	Urea (mg/dL)
Pretreatment	Control		0.70 ± 0.02^a^	1.50 ± 0.14^a^	4.43 ± 0.43^ab^
	7 days	DMBA	1.15 ± 0.06^b^	2.15 ± 0.23^cd^	5.78 ± 0.40^cd^
		HaE	0.98 ± 0.01^b^	1.67 ± 0.14^abc^	4.61 ± 0.30^ab^
	14 days	DMBA	2.17 ± 0.15^d^	2.69 ± 0.18^e^	5.54 ± 0.14^cd^
		HaE	1.15 ± 0.05^b^	1.30 ± 0.17^a^	4.88 ± 0.22^b^

Posttreatment	Control		0.73 ± 0.02^a^	1.51 ± 0.15^a^	4.39 ± 0.43^ab^
	7 days	DMBA	1.20 ± 0.06^b^	2.07 ± 0.18^bcd^	5.06 ± 0.09^cd^
		HaE	0.97 ± 0.01^b^	1.42 ± 0.14^a^	5.06 ± 0.09^cd^
	14 days	DMBA	1.85 ± 0.12^c^	2.45 ± 0.09^de^	6.15 ± 0.05^d^
		HaE	1.16 ± 0.05^b^	1.59 ± 0.12^ab^	4.49 ± 0.45^ab^

Values are given as mean ± SEM for 6 rats in each group.

Each value not sharing a common letter superscript is significantly different (*P* < 0.05).

**Table 4:** Modulatory influence of *Holothuria atra* extract (HaE) on liver, malondialdehyde (MDA), and reduced glutathione (GSH) levels of 7,12-dimethylbenz[a]anthracene (DMBA) treated rats.

Treatment	Groups		MDA (nmol/g tissue)	GSH (mg/g tissue)
Pretreatment	Control		3.24 ± 0.04^a^	3.90 ± 0.54^c^
	7 days	DMBA	3.44 ± 0.07^ab^	1.43 ± 0.31^a^
		HaE	3.46 ± 0.04^ab^	1.98 ± 0.30^ab^
	14 days	DMBA	4.79 ± 0.53^cd^	1.35 ± 0.10^a^
		HaE	3.50 ± 0.12^ab^	1.66 ± 0.27^b^

Posttreatment	Control		3.21 ± 0.04^a^	3.81 ± 0.57^c^
	7 days	DMBA	4.12 ± 0.36^bc^	2.90 ± 0.16^d^
		HaE	4.03 ± 0.13^b^	1.58 ± 0.05^a^
	14 days	DMBA	5.05 ± 0.25^d^	1.39 ± 0.12^a^
		HaE	3.95 ± 0.12^ab^	2.14 ± 0.09^b^

Values are given as mean ± SEM for 6 rats in each group.

Each value not sharing a common letter superscript is significantly different (*P* < 0.05).

**Table 5:** Modulatory influence of *Holothuria atra* extract (HaE) on some liver antioxidant enzymes activities of 7,12-dimethylbenz[a]anthracene (DMBA) treated rats.

Treatment	Groups		GST (nmol/min/g tissue)	SOD (U/g tissue)	CAT (U/min)
Pretreatment	Control		2.70 ± 0.49^a^	264.08 ± 28.23^d^	686.40 ± 73.64^d^
	7 days	DMBA	1.51 ± 0.18^b^	201.53 ± 13.72^b^	473.6 ± 55.50^ab^
		HaE	1.45 ± 0.08^b^	183.21 ± 9.71^ab^	448.00 ± 14.96^ab^
	14 days	DMBA	1.13 ± 0.16^b^	168.32 ± 2.48^ab^	362.40 ± 41.83^a^
		HaE	2.18 ± 0.24^a^	214.53 ± 6.87^c^	560.00 ± 65.72^bc^

Posttreatment	Control		2.68 ± 0.49^a^	268.14 ± 28.84^d^	683.40 ± 73.68^d^
	7 days	DMBA	1.63 ± 0.24^b^	166.39 ± 12.81^ab^	472.00 ± 32.00^ab^
		HaE	1.54 ± 0.10^b^	164.14 ± 11.81^ab^	552.00 ± 38.78^bc^
	14 days	DMBA	1.20 ± 0.13^b^	146.14 ± 7.96^a^	440.00 ± 17.88^ab^
		HaE	2.21 ± 0.10^a^	220.10 ± 2.87^c^	584.00 ± 29.93^c^

Values are given as mean ± SEM for 6 rats in each group.

Each value not sharing a common letter superscript is significantly different (*P* < 0.05).

## References

[1] Li Z., Romanoff L. C., Trinidad D. A. (2014). Quantification of 21 metabolites of methylnaphthalenes and polycyclic aromatic hydrocarbons in human urine. *Analytical and Bioanalytical Chemistry*.

[2] Krüger O., Kalbe U., Meißner K., Sobottka S. (2014). Sorption effects interfering with the analysis of polycyclic aromatic hydrocarbons (PAH) in aqueous samples. *Talanta*.

[3] Chen Y., Feng Y., Xiong S. (2011). Polycyclic aromatic hydrocarbons in the atmosphere of Shanghai, China. *Environmental Monitoring and Assessment*.

[4] Katz I. S. S., Albuquerque L. L., Suppa A. P. (2014). 7,12-dimethylbenz(a)anthracene-induced myelotoxicity differs in mice selected for high or low acute inflammatory response: relationship with aryl hydrocarbon receptor polymorphism. *International Journal of Toxicology*.

[5] Kumar R., Kaur R., Singh A. P., Arora S. (2014). Diminution of hepatic response to 7, 12-dimethylbenz(*α*)anthracene by ethyl acetate fraction of *Acacia catechu* Willd. through modulation of xenobiotic. *PLoS ONE*.

[6] Nandakumar N., Balasubramanian M. P. (2012). Hesperidin a citrus bioflavonoid modulates hepatic biotransformation enzymes and enhances intrinsic antioxidants in experimental breast cancer rats challenged with 7, 12- Dimethylbenz (a) anthracene. *Journal of Experimental Therapeutics and Oncology*.

[7] Bharali R., Tabassum J., Azad M. R. H. (2003). Chemomodulatory effect of moringa oleifera, lam, on hepatic carcinogen metabolising enzymes, antioxidant parameters and skin papillomagenesis in mice. *Asian Pacific Journal of Cancer Prevention*.

[8] Paliwal R., Sharma V., Sharma S., Yadav S. (2011). Anti-nephrotoxic effect of administration of *Moringa oleifera* Lam in amelioration of DMBA-induced renal carcinogenesis in Swiss albino mice. *Biology and Medicine*.

[9] Rastogi S., Shukla Y., Paul B. N., Chowdhuri D. K., Khanna S. K., Das M. (2007). Protective effect of *Ocimum sanctum* on 3-methylcholanthrene, 7,12-dimethylbenz(a)anthracene and aflatoxin B1 induced skin tumorigenesis in mice. *Toxicology and Applied Pharmacology*.

[10] Chattopadhyay R. R. (2003). Possible mechanism of hepatoprotective activity of *Azadirachta indica* leaf extract: part II. *Journal of Ethnopharmacology*.

[11] Koyama T., Chounan R., Uemura D., Yamaguchi K., Yazawa K. (2006). Hepatoprotective effect of a hot-water extract from the edible thorny oyster *Spondylus varius* on carbon tetrachloride-induced liver injury in mice. *Bioscience, Biotechnology and Biochemistry*.

[12] Zaki M. A. (2005). Effects of the crude toxin of sea cucumbers *Holothuria atra* on some hematological and biochemical parameters in rats. *Egyptian Journal of Natural Toxins*.

[13] Ridzwan B. H., Leong T. C., Idid S. Z. (2003). The antinociceptive effects of water extracts from sea cucumbers *Holothuria leucospilota Brandt*, *Bohadschia marmorata vitiensis Jaeger* and coelomic fluid from *Stichopus hermanii*. *Pakistan Journal of Biological Sciences*.

[14] Hamel J.-F., Mercier A., Lovatelli A., Conand C., Purcell S., Uthicke S., Hamel J.-F., Mercier A. (2004). Synchronous gamete maturation and reliable spawning induction method in holothurians. *Advances in Sea Cucumber Aquaculture and Management*.

[15] Esmat A. Y., Said M. M., Soliman A. A., El-Masry K. S. H., Badiea E. A. (2013). Bioactive compounds, antioxidant potential, and hepatoprotective activity of sea cucumber (*Holothuria atra*) against thioacetamide intoxication in rats. *Nutrition*.

[16] Hasan M. H. (2009). Stock assessment of holothuroid populations in the Red Sea waters of Saudi Arabia. *SPC Beche -de-mer Information Bulletin*.

[17] Purcell S. W., Samyn Y., Conand C. (2012). *Commercially Important Sea Cucumbers of the World*.

[18] Yasumoto T., Nakamura K., Hashimoto Y. (1967). A new saponinholothurin isolated from the sea cucumber *Holothuria vagabunda*. *Agricultural Biolology and Chemistry*.

[19] Sanchez-Moreno C., Larrauri J. A., Saura-Calixto F. (1998). A procedure to measure the antiradical efficiency of polyphenol. *Journal of the Science of Food and Agriculture*.

[20] van den Heuvel M. J., Clark D. G., Fielder R. J. (1990). The international validation of a fixed-dose procedure as an alternative to the classical LD_50_ test. *Food and Chemical Toxicology*.

[21] Whitehead A., Curnow R. N. (1992). Statistical evaluation of the fixed-dose procedure. *Food and Chemical Toxicology*.

[22] Reitman S., Frankel S. (1957). A colorimetric method for the determination of serum glutamic oxalacetic and glutamic pyruvic transaminases. *The American Journal of Clinical Pathology*.

[23] Szasz G. (1974). New substrates for measuring gamma-glutamyl transpeptidase activity. *Zeitschrift für klinische Chemie und klinische Biochemie*.

[24] Tietz N. W., Burtis C. A., Ashwood E. R. (1994). *Tietz Textbook of Clinical Chemistry*.

[25] Belfield A., Goldberg D. M. (1971). Revised assay for serum phenyl phosphatase activity using 4-amino-antipyrine.. *Enzyme*.

[26] Walters M. I., Gerarde H. W. (1970). An ultramicromethod for the determination of conjugated and total bilirubin in serum or plasma. *Microchemical Journal*.

[27] Tietz N. W., Andresen B. D. (1986). *Textbook of Clinical Chemistry*.

[28] Tietz N. W., Finley P., Pruden E., Amerson A. (1990). *Clinical Guide to Laboratory Tests Saunders*.

[29] Ohkawa H., Ohishi N., Yagi K. (1979). Assay for lipid peroxides in animal tissues by thiobarbituric acid reaction. *Analytical Biochemistry*.

[30] Beutler E., Duron O., Kelly B. M. (1963). Improved method for the determination of blood glutathione. *The Journal of Laboratory and Clinical Medicine*.

[31] Aebi H. (1984). Catalase *in vitro*. *Methods in Enzymology*.

[32] Habig W. H., Pabst M. J., Jakoby W. B. (1974). Glutathione S- transferases: the first enzymatic step in mercapturic acid formation. *The Journal of Biological Chemistry*.

[33] Nishikimi M., Appaji Rao N., Yagi K. (1972). The occurrence of superoxide anion in the reaction of reduced phenazine methosulfate and molecular oxygen. *Biochemical and Biophysical Research Communications*.

[34] Koul A., Arora N., Tanwar L. (2010). Lycopene mediated modulation of 7,12 dimethlybenz (A) anthracene induced hepatic clastogenicity in male Balb/c mice. *Nutricion Hospitalaria*.

[35] Al-Athar A. M. (2004). The influence of dietary grape seed oil on DMBA-induced liver enzymes disturbances in the frog, *Rana ridibunda*. *Pakistan Journal of Nutrition*.

[36] Digiovanni J., Juchau M. R. (1980). Biotransformation and bioactivation of 7,12-dimethylbenz[a]anthracene (7,12-DMBA). *Drug Metabolism Reviews*.

[37] Zhang Y., Talalay P., Cho C.-G., Posner G. H. (1992). A major inducer of anticarcinogenic protective enzymes from broccoli: isolation and elucidation of structure. *Proceedings of the National Academy of Sciences of the United States of America*.

[38] Zhou Q.-M., Wang X.-F., Liu X.-J. (2011). Curcumin improves MMC-based chemotherapy by simultaneously sensitising cancer cells to MMC and reducing MMC-associated side-effects. *European Journal of Cancer*.

[39] Salama S. M., Abdulla M. A., AlRashdi A. S., Ismail S., Alkiyumi S. S., Golbabapour S. (2013). Hepatoprotective effect of ethanolic extract of *Curcuma longa* on thioacetamide induced liver cirrhosis in rats. *BMC Complementary and Alternative Medicine*.

[40] Schwartsmann G. (2000). Marine organisms and other novel natural sources of new cancer drugs. *Annals of Oncology*.

[41] Schwartsmann G., da Rocha A. B., Berlinck R. G. S., Jimeno J. (2001). Marine organisms as a source of new anticancer agents. *The Lancet Oncology*.

[42] Althunibat O. Y., Hashim R. B., Taher M., Daud J. M., Ikeda M.-A., Zali B. I. (2009). *In vitro* antioxidant and antiproliferative activities of three Malaysian sea cucumber species. *European Journal of Scientific Research*.

[43] Xu Y., Chen J., Yu X. (2010). Protective effects of chlorogenic acid on acute hepatotoxicity induced by lipopolysaccharide in mice. *Inflammation Research*.

[44] Vijayabaskaran M., Yuvaraja K. R., Babu G., Sivakumar P., Perumal P., Jayakar B. (2010). Hepatoprotective and antioxidant activity of *Symplocos racemosa* bark extract on DMBA induced hepatocellular carcinoma in rats. *International Journal of Current Trends in Science and Technology*.

[45] El Kholy W., Serag H., Zakaria A., El Metwaly A. (2013). The Potency of some natural products on dimethyl benz (a) antheracene (DMBA) induced hepatotoxicity in rats. *The Egyptian Journal of Hospital Medicine*.

[46] Ahmed M. O., Mohamed M. P., Mahmoud M. A. (2014). Curcumin and naringin prevent 7,12-dimethylbenz(a)anthracene-induced hepatic injury by suppressing inflammation and oxidative stress. *Journal of International Academic Research for Multidisciplinary*.

[47] Farag-Allah A. M., Abdel-Dayem S. M. (2001). Biochemical, histological and ultrastructural studies on the protective effect of vitamin A against the carcinogenic effect of 7, 12-dimethylbenz(*a*)anthracene (DMBA) on the liver of albino rats. *Egyptian Journal of Zoology*.

[48] Ali D. A., Ismail M. F., Badr A. H. (2013). Hepatoprotective effect of ginger extract against the toxicity of 7, 12-dimethylbenz (a) anthracene (DMBA) in albino rats. *World Journal of Pharmaceutical Sciences*.

[49] Bandyopadhyay U., Das D., Banerjee R. K. (1999). Reactive oxygen species: oxidative damage and pathogenesis. *Current Science*.

[50] Sharma V., Paliwal R., Janmeda P., Sharma S. (2012). Chemopreventive efficacy of Moringa oleifera pods against 7, 12-dimethylbenz[a]anthracene induced hepatic carcinogenesis in mice. *Asian Pacific Journal of Cancer Prevention*.

[51] Moore C. J., Tricomi W. A., Gould M. N. (1986). Interspecies comparison of polycyclic aromatic hydrocarbon metabolism in human and rat mammary epithelial cells. *Cancer Research*.

[52] Muto T., Takasaki S., Takahashi H. (2003). Initial changes of hepatic glycogen granules and glycogen phosphorylase a after exposure to 7, 12-dimethylbenz[a] anthracene in rats. *Journal of Toxicologic Pathology*.

[53] Parmar J., Sharma P., Verma P., Goyal P. K. (2010). Chemopreventive action of *Syzygium cumini* on DMBA-induced skin papillomagenesis in mice. *Asian Pacific Journal of Cancer Prevention*.

[54] Parmar J., Verma P., Sharma P., Goyal P. K. (2011). Elimination of deleterious effects of DMBA-induced skin carcinogenesis in mice by *Syzygium cumini*seed extract. *Integrative Cancer Therapies*.

[55] Pushpakiran G., Mahalakshmi K., Anuradha C. V. (2004). Taurine restores ethanol-induced depletion of antioxidants and attenuates oxidative stress in rat tissues. *Amino Acids*.

[56] Deleve L. D., Wang X., Kuhlenkamp J. F., Kaplowitz N. (1996). Toxicity of azathioprine and monocrotaline in murine sinusoidal endothelial cells and hepatocytes: the role of glutathione and relevance to hepatic venoocclusive disease. *Hepatology*.

[57] Singh H., Bedi P. S., Singh B. (2011). Hepatoprotective activity of turmeric and garlic against 7-12, dimethylbenzanthracene induced liver damage in Wistar albino rats. *European Journal of Medicinal Plants*.

[58] Gaté L., Schultz M., Walsh E. (1998). Impact of dietary supplement of *Crassostrea gigas* extract (JCOE) on glutathione levels and glutathione S-transferase activity in rat tissues. *In Vivo*.

[59] Fahmy S. R., Hamdi S. A. H. (2011). Curative effect of the Egyptian marine *Erugosquilla massavensis* extract on carbon tetrachloride-induced oxidative stress in rat liver and erythrocytes. *European Review for Medical and Pharmacological Sciences*.

[60] Park S.-W., Lee C.-H., Yeong S. K. (2008). Protective effect of baicalin against carbon tetrachloride-induced acute hepatic injury in mice. *Journal of Pharmacological Sciences*.

[61] Koul A., Mohan V., Bharati S. (2014). *Azadirachta indica* mitigates DMBA-induced hepatotoxicity: a biochemical and radiometric study. *Indian Journal of Biochemistry and Biophysics*.

[62] Lakshmi A., Subramanian S. (2014). Chemotherapeutic effect of tangeretin, a polymethoxylated flavone studied in 7, 12-dimethylbenz(a)anthracene induced mammary carcinoma in experimental rats. *Biochimie*.

[63] Cerutti P., Ghosh R., Oya Y., Amstad P. (1994). The role of the cellular antioxidant defense in oxidant carcinogenesis. *Environmental Health Perspectives*.

[64] Suhail N., Bilal N., Hasan S., Banu N. (2011). Chronic unpredictable stress exacerbates 7,12-dimethylbenz (a) anthracene induced hepatotoxicity and nephrotoxicity in Swiss albino mice. *Molecular and Cellular Biochemistry*.

[65] Bedi P. S., Priyanka S. (2012). Effects of garlic against 7-12, Dimethyl benzanthracene induced toxicity in Wistar albino rats. *Asian Journal of Pharmaceutical and Clinical Research*.

